# The Interplay of Cancer and Hypertension: Rising Mortality and Widening Disparities Across the United States (1999–2023)

**DOI:** 10.3390/medicina61050917

**Published:** 2025-05-19

**Authors:** Ibrahim Ali Nasser, Shereen Asghar, Laraib Masud, Muhammad Ali Hafeez, Sonia Hurjkaliani, Eeshal Zulfiqar, Maryam Shahzad, Husain Ahmed, Shahrukh Khan, Sajeel Ahmed, Qadeer Abdul, Muhammed Ameen Noushad, Rabia Nusrat, Sana Azhar, Charles Dominic Ward, Mushood Ahmed, Raheel Ahmed

**Affiliations:** 1 South Tyneside and Sunderland NHS Foundation Trust, Sunderland SR4 7TP, UK; i.nasser@live.co.uk (I.A.N.); icedive111@hotmail.com (S.A.); srkzrk@gmail.com (S.K.); sana14393@gmail.com (S.A.); 2 Islamabad Medical and Dental College, Islamabad 44000, Pakistan; laraibmasud1@gmail.com; 3 University Hospital of North Tees, Stockton-on-Tees TS19 8PE, UK; drmahalihafeez@gmail.com; 4 Department of Medicine, Dow University of Health Sciences, Karachi 74200, Pakistan; shurjkaliani@gmail.com (S.H.); eeshalzulfiqar12@gmail.com (E.Z.); maryamshahzad575@gmail.com (M.S.); 5 South Tees Foundation Trust, Middlesbrough TS4 3BW, UK; hussain_ahmed17@hotmail.com (H.A.); sajeel22@hotmail.com (S.A.); 6 Darrent Valley Hospital, Dartford and Gravesham NHS Trust, Dartford DA2 8DA, UK; qadeer.abdul16@hotmail.com; 7 University Hospitals Plymouth NHS Trust, Plymouth PL68DH, UK; muhammedameen.noushad@nhs.net; 8 Southend University Hospital NHS Foundation Trust, Southend-on-Sea SS0 0RY, UK; rabianusrat@yahoo.com; 9 Catterick Medical Centre, Ministry of Defence, Catterick DL9 3PZ, UK; cdward6697@gmail.com; 10 Department of Medicine, Rawalpindi Medical University, Rawalpindi 46000, Pakistan; mushood07@gmail.com; 11 Royal Brompton Hospital, London SW3 6NP, UK; 12 National Heart & Lung Institute, Imperial College London, London SW3 6LY, UK

**Keywords:** hypertension, cancer, mortality, United States

## Abstract

*Background and Objectives*: Growing evidence suggests a strong relationship between hypertension and cancer, which can increase the risk of poor prognosis. However, data regarding mortality related to cancer and hypertension are limited. Our study aims to analyze the mortality trends related to cancer and hypertension in the United States from 1999 to 2023. *Materials and Methods*: A retrospective observational analysis was conducted using mortality data for the adult U.S. population from 1999 to 2023, retrieved from the CDC WONDER database using death certificates. Age-adjusted mortality rates (AAMRs) were calculated, and annual percentage changes (APCs) were analyzed using JoinPoint Regression. *Results*: From 1999 to 2023, a total of 1,406,107 deaths related to cancer and hypertension were recorded in the United States. The AAMR increased from 12.59 in 1999 to 35.49 in 2023. Males had a higher mortality rate compared to women throughout the study period (AAMR; 30.3 vs. 20.4). Non-Hispanic (NH) Black Americans, or African Americans had the highest mortality rates, followed by NH white, Hispanic or Latino groups, and other NH groups. The highest AAMR was observed in the South, followed by the Midwest, the Northeast, and the West. Rural areas had higher mortality rates compared to urban areas. *Conclusions*: Cancer- and hypertension-related mortality rates have consistently increased in the United States from 1999 to 2023, particularly affecting males, NH Black Americans, the southern region, and rural areas. The trends highlight the need for targeted prevention, including early screening, lifestyle changes, and treatment adherence.

## 1. Introduction

Hypertension is becoming more prevalent in the general population and is a major health problem [1], substantially contributing to cardiovascular disease and death despite advances in medical treatment [2,3]. It is believed that about 32–47% of the population suffers from hypertension [4,5]. Cancer is the second leading cause of mortality, and approximately 19.3 million new cancer cases were diagnosed, with an estimated 10 million cancer-related deaths recorded in the year 2020 [6]. In the United States, around 53.6% of patients hospitalized with cancer have concomitant hypertension, which can worsen the prognosis [7].

Recent studies have suggested a correlation between the increased incidence of cancers and risk factors such as hypertension, type II diabetes, obesity, smoking, and a sedentary lifestyle [8,9]. Jain and Town have established a significant association between cancer and hypertension; they have estimated that about 37% of cancer patients have underlying hypertension [10,11]. The Childhood Cancer Survivor Study has suggested that hypertension is more prevalent in individuals with a background of cancer [12].

The association between hypertension and cancer has been well-researched. The significance of managing both conditions adequately is of prime importance, as hypertension can influence the management of cancer due to the risk of chemotherapy-induced cardiotoxicity and even the discontinuation of certain cancer therapies [11,13,14]. Over the past few years, growing evidence has highlighted hypertension’s dual role in oncology. Not only is hypertension a common side effect of several cancer therapies, particularly angiogenesis inhibitors like VEGF inhibitors, but it also serves as a significant risk factor for cancer therapy-related cardiotoxicity. Elevated blood pressure can exacerbate vascular damage and accelerate cardiac dysfunction, compounding the cardiovascular risks posed by treatments such as anthracyclines and targeted agents. As a result, the early identification and management of hypertension have become crucial components of cardio-oncology care to mitigate long-term cardiovascular complications in cancer survivors [15,16].

A fine grasp of the correlation between hypertension and cancer is the key to optimizing therapies for high-risk cohorts to ameliorate the mortality rates influenced by hypertension and cancer. This study examined data from the CDC WONDER database spanning 1999 to 2023 to assess mortality trends among adults with hypertension and cancer in the United States based on gender, ethnic backgrounds, and regional variation.

## 2. Materials and Methods

### 2.1. Study Setting and Population

This study analyzed hypertension and cancer-related mortality across the United States using data from the CDC WONDER (Centers for Disease Control and Prevention Wide-Ranging Online Data for Epidemiologic Research) database. The database includes comprehensive death certificate data from all 50 states and the District of Columbia and follows a retrospective observational study design.

Mortality from hypertension was identified using ICD-10 codes I10–I15, while cancer-related deaths were classified under ICD-10 codes C00–D48. The analysis encompassed people aged 25 years and older. Since the study used publicly available, de-identified government data, institutional review board approval was not required. To ensure methodological rigor, the study adhered to the STROBE guidelines [17].

### 2.2. Data Collection

Data on hypertension- and cancer-related deaths, population sizes, and demographic characteristics (age, sex, race/ethnicity, and regional details) were collected from 1999 to 2023. The racial and ethnic groups analyzed included non-Hispanic (NH) white, NH Black or African Americans and other NH groups (e.g., NH Asian or Pacific Islanders, NH American Indians or Alaska Natives), and Hispanic or Latino populations. These classifications align with previous studies utilizing CDC WONDER data derived from death certificates [18,19,20,21]. Age stratification was performed, dividing age into the following categories: 25–34, 35–44, 45–54, 55–64, 65–74, 75–84, and 85 years and older.

Further analysis of mortality trends was conducted by state, U.S. Census regions (Northeast, Midwest, South, and West), and levels of county urbanization. Counties were classified as rural (including micropolitan and noncore areas) or urban (large central metro, large fringe metro, medium metro, and small metro areas) based on the 2013 Urban-Rural Classification Scheme from the National Center for Health Statistics [22].

### 2.3. Statistical Analysis

Crude mortality rates (CMRs) and age-adjusted mortality rates (AAMRs) were calculated per 100,000 individuals. CMRs were determined by dividing the total number of deaths related to hypertension and cancer by the population for each respective year. AAMRs were calculated by standardizing death counts to the U.S. population structure in the year 2000, enabling consistent comparisons across years [4].

The Joinpoint Regression Program (Joinpoint version 5.1.0, National Cancer Institute) was employed to analyze mortality trends [23]. This software detects significant shifts in AAMRs and CMRs over time using log-linear regression models applied to periods of change. Annual percentage changes (APCs) and corresponding 95% confidence intervals (CIs) were calculated to assess these trends, with joinpoints marking periods of significant variation. The Monte Carlo permutation test was used to assess statistical significance, with APCs considered significant if the slope differed significantly from zero in a two-tailed t-test. Statistical significance was set at *p* < 0.05.

## 3. Results

### 3.1. Overall

From 1999 to 2023, the United States recorded 1,406,107 deaths attributed to hypertension (HTN) and cancer. The AAMR increased from 12.59 in 1999 to 35.49 in 2023. The AAMR experienced a significant rise from 12.59 in 1999 to 19.06 in 2001, with an APC of 21.05* (13.18 to 26.63, *p* < 0.001). This upward trend continued from 19.06 in 2001 to 23.91 in 2007, with an APC of 3.01* (1.36 to 5.73, *p* = 0.0036). From 2007 to 2018, the AAMR remained relatively stable. However, the rate rose again from 25.55 in 2018 to 34.69 in 2021, showing an APC of 12.18* (9.05 to 14.23, *p* = 0.0036). A period of stability was observed from 2021 to 2023 (Table S1).

### 3.2. HTN- and Cancer-Related AAMR Stratified by Sex

Throughout the study period, males consistently exhibited a higher AAMR. For males, their AAMR rose from 14.52 in 1999 to 22.92 in 2001, with an APC of 22.54* (13.18 to 29.25, *p* < 0.001). The rate continued to increase from 22.92 in 2001 to 29.5 in 2007, with an APC of 3.23* (1.56 to 6.03, *p* = 0.0040). From 2007 to 2018, the AAMR remained stable. However, it surged again from 32.27 in 2018 to 43.64 in 2021, showing an APC of 12.00* (8.92 to 14.06, *p* = 0.0024), followed by stability from 2021 to 2023.

**Figure 1:**
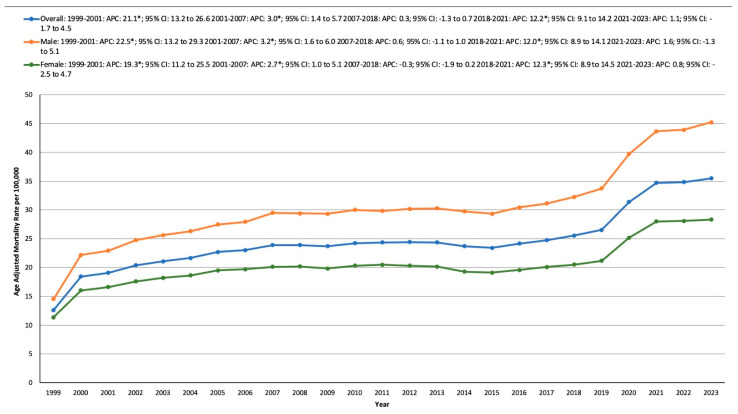
Overall and sex-specific, age-adjusted mortality rates per 100,000 in the population of the United States, 1999–2023, * indicates statistical significance.

For females, the AAMR rose from 11.35 in 1999 to 16.59 in 2001, with an APC of 19.25* (11.23 to 25.45, *p* < 0.001). The rate then grew from 16.59 in 2001 to 20.13 in 2007, reflecting an APC of 2.66* (0.97 to 5.09, *p* = 0.0068). There was stability from 2007 to 2018, followed by a rise from 20.52 in 2018 to 27.98 in 2021, showing an APC of 12.29* (8.88 to 14.51, *p* = 0.0080). This was followed by another period of stability from 2021 to 2023 (Tables S2 and S3, Figure 1).

### 3.3. HTN- and Cancer-Related AAMR Stratified by Race/Ethnicity

Throughout the study period, the AAMR was observed in the NH Black or African American population, followed by the NH white, Hispanic or Latino populations, and other NH groups.

For the NH Black or African American group, the AAMR rose from 26.51 in 1999 to 36.65 in 2001, with an APC of 16.86* (8.12 to 24.64, *p* < 0.001). The rate remained stable during the periods 2001–2010, 2010–2018, 2018–2021, and 2021–2023.

In the NH white population, the AAMR increased significantly from 11.42 in 1999 to 17.7 in 2001, with an APC of 22.33* (13.52 to 28.30, *p* < 0.001). It continued to rise from 17.7 in 2001 to 22.87 in 2007 (APC: 3.23*; 1.65 to 5.99, *p* = 0.003). After a period of stability from 2007 to 2018, the AAMR rose again, from 25.3 in 2018 to 35.09 in 2021, with an APC of 13.12* (9.87 to 15.22, *p* = 0.002), followed by stability from 2021 to 2023.

The Hispanic or Latino group experienced an increase in AAMR from 8.76 in 1999 to 17.36 in 2005, with an APC of 8.41* (3.89 to 16.59, *p* = 0.024). This was followed by stability in the periods 2005–2018, 2018–2021, and 2021–2023.

**Figure 2:**
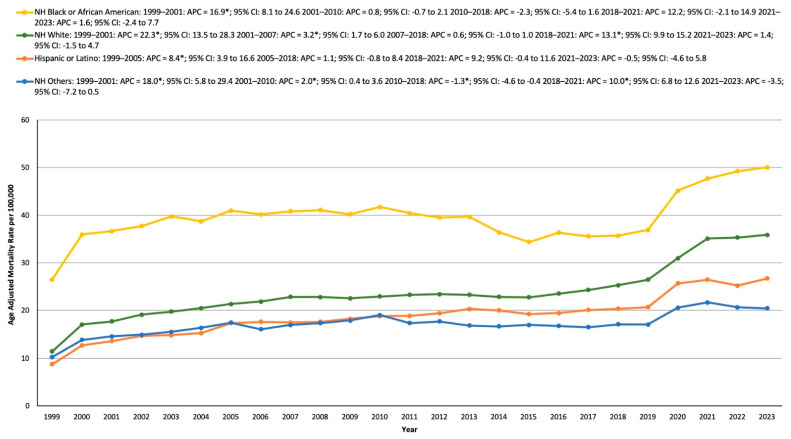
Age-adjusted mortality rates per 100,000 in the population stratified by race/ethnicity in the United States, 1999–2023, * indicates statistical significance.

Finally, in the other NH populations group, the AAMR increased from 10.27 in 1999 to 14.56 in 2001, with an APC of 17.98* (5.78 to 29.39, *p* < 0.001). It continued to rise from 14.56 in 2001 to 19.04 in 2010 (APC: 2.02*; 0.39 to 3.61, *p* = 0.028). After a stable period from 2010 to 2018, the AAMR increased again from 17.09 in 2018 to 21.68 in 2021, with an APC of 10.04* (6.78 to 12.57, *p* = 0.006), followed by stability from 2021 to 2023 (Table S4, Figure 2).

### 3.4. HTN- and Cancer-Related AAMR Stratified by Geographical Region

#### 3.4.1. Statewide

Throughout the study period, significant statewide variation in HTN- and cancer-related mortality was observed. From 1999 to 2020, states falling within the top 90th percentile for mortality rates included Mississippi, Oklahoma, the District of Columbia, Nebraska, and Ohio, while those in the bottom 10th percentile were Utah, Nevada, Arizona, Massachusetts, and Florida. In the subsequent period from 2021 to 2023, the states with the highest mortality rates were Oklahoma, Mississippi, Nebraska, South Carolina, and Minnesota, whereas Utah, Connecticut, Massachusetts, Alaska, and Illinois ranked in the lowest 10th percentile (Table S5).

#### 3.4.2. Census Region

From 1999 to 2023, the highest HTN- and cancer-related mortality rates were observed in the South, followed by the Midwest, the Northeast, and finally, the West.

In the Northeast, the AAMR rose from 12.56 in 1999 to 18.27 in 2001, with an APC of 18.16* (6.58 to 25.40; *p* < 0.001). It continued to increase from 18.27 in 2001 to 23.33 in 2011 (APC: 1.87*; 0.42 to 3.49; *p* = 0.0312). However, a significant decline followed, with the AAMR dropping from 23.33 in 2011 to 20.11 in 2016 (APC: −3.00*; −6.93 to −0.51; *p* = 0.0296). Subsequently, the rate climbed again, reaching 26.4 in 2023, with an APC of 4.90* (3.48 to 6.97; *p* = 0.0060).

In the southern region, the AAMR increased dramatically from 11.11 in 1999 to 18.01 in 2001, with an APC of 28.51* (13.52 to 38.88; *p* < 0.001). This upward trend continued, with the rate rising further from 18.01 in 2001 to 27.3 in 2017 (APC: 1.84*; 95%CI: 1.18 to 2.34; *p* < 0.001). The AAMR then surged from 27.3 in 2017 to 43.77 in 2023, exhibiting an APC of 9.37* (7.83 to 11.51; *p* < 0.001).

In the midwest, the AAMR grew from 14.22 in 1999 to 21.06 in 2001, with an APC of 20.15* (12.16 to 26.19; *p* < 0.00001). This was followed by an additional increase from 21.06 in 2001 to 27.07 in 2007 (APC: 3.59*; 1.79 to 5.46; *p* = 0.0028). However, the rate declined from 27.07 in 2007 to 24.03 in 2018 (APC: −1.26*; −2.07 to −0.78; *p* = 0.0016). It then rose sharply from 24.03 in 2018 to 33.32 in 2021, with an APC of 11.97* (8.60 to 14.04; *p* = 0.0020), remaining stable from 2021 to 2023.

**Figure 3:**
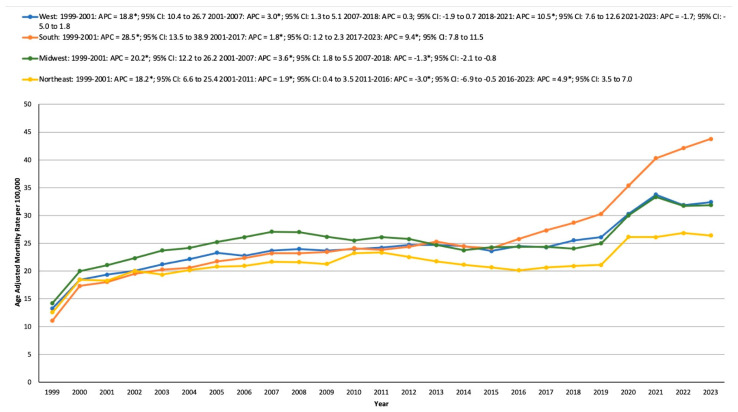
Age-adjusted mortality rates per 100,000 in the population by U.S. Census region, 1999–2023, * indicates statistical significance.

Lastly, in the west, the AAMR increased significantly from 13.27 in 1999 to 19.32 in 2001, with an APC of 18.76* (10.43 to 26.66; *p* < 0.001). The rate continued to climb from 19.32 in 2001 to 23.64 in 2007 (APC: 3.00*; 1.27 to 5.12; *p* = 0.0040). Stability was observed from 2007 to 2018, followed by a significant increase from 25.5 in 2018 to 33.75 in 2021, with an APC of 10.51* (7.62 to 12.56; *p* = 0.0004), remaining stable through 2023 (Table S6, Figure 3).

#### 3.4.3. Urban and Rural Areas

**Figure 4:**
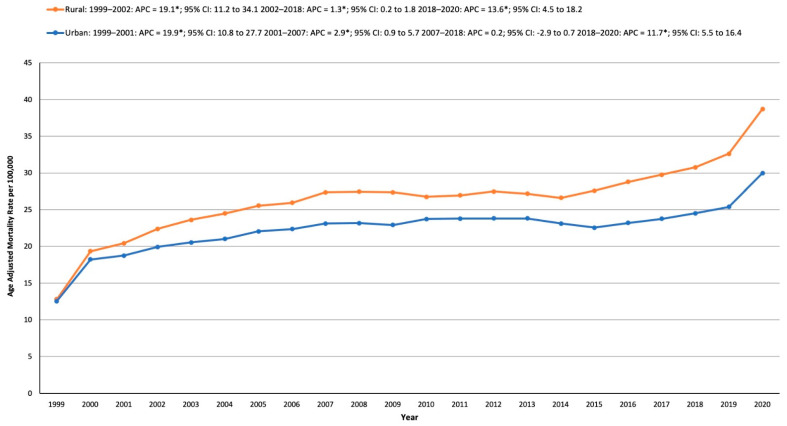
Age-adjusted mortality rates per 100,000 in the population by urbanization in the United States, 1999–2020. * Data for urbanization AAMRs were unavailable for 2021–2023.

Throughout the study period, rural areas consistently recorded a significantly higher AAMR than urban areas. In urban regions, the AAMR saw a sharp rise from 12.53 in 1999 to 18.75 in 2001, with an APC of 19.86* (10.83 to 27.74, *p* < 0.001). This was followed by a more gradual increase from 18.75 in 2001 to 23.1 in 2007 (APC: 2.92*; 0.92 to 5.70, *p* = 0.012). The rates then remained stable between 2007 and 2018 before increasing again from 24.51 in 2018 to 29.99 in 2020 (APC: 11.73*; 5.47 to 16.40, *p* < 0.001). In rural areas, the AAMR initially climbed significantly from 12.79 in 1999 to 22.38 in 2002, with an APC of 19.05* (11.16 to 34.07, *p* < 0.001). A slower but steady increase followed, as the rate rose from 22.38 in 2002 to 30.76 in 2018 (APC: 1.26*; 0.19 to 1.76, *p* = 0.033). This was succeeded by a marked surge from 30.76 in 2018 to 38.71 in 2020, with an APC of 13.58* (4.48 to 18.19, *p* < 0.001) (Table S7, Figure 4).

### 3.5. HTN- and Cancer-Related AAMR Stratified by Ten-Year Age Groups

In the age-stratified analysis, the highest CMRs were recorded in the 85+ years and 75–84 years age groups, followed by the 25–34, 35–44, and 45–54 years age groups. In contrast, the 65–74 and 55–64-year age groups exhibited the lowest CMRs (Table S8).

## 4. Discussion

Overall, from the data compiled, there were 1,406,107 deaths attributed to both hypertension and cancer in the United States from the period 1999 to 2023. The overall prevalence from 1999 to 2023 of deaths pertaining to cancer and hypertension has increased, from its lowest at 22,209 to its highest in 2023, with 98,581 deaths in the country. Males had higher AAMRs compared to women for all periods. Ethnic minority groups had higher AAMRs compared to the white population.

Multiple studies have shown that hypertension is a risk factor for developing various cancers, such as renal cell carcinomas [24,25], and is a co-morbidity increasing mortality in these patients. Hypertension is a disease where there is dysregulation of the cardiovascular system, with stiffer vessels and a heart that has to undergo increased strain to pump blood through the higher resistance system. This will lead to further complications in the vascular system, such as aneurysms [26], myocardial ischemia and infarction, strokes, other forms of reduced perfusion to the liver, and renal systems, which carry high mortality.

With regard to differences in sex and the age-adjusted rate of mortality, males persistently had a higher rate of death than females. One explanation is due to the higher prevalence of hypertension in males in the US population versus females [27]. Globally, some studies suggest that hypertension awareness is higher among the female population compared to the male population, making it more likely for women to check for higher blood pressure, seek treatment, and adhere to treatment [27,28]. The above finding was also identified in the NCHS Data Brief No 511, where adult US women aged 18–39 had increased awareness of their hypertension status and were more likely to be undergoing treatment. Ultimately, this leads to the increased control of hypertension in women, with 22.8% overall control vs. men, with 18.9% control overall, with a greater difference seen in the age group 40–59, with 23.2% compared to 13.6% overall control, respectively [29].

Key findings from the National Health and Nutrition Examination Survey demonstrated that 51% of male US adults aged 18 and over versus 39.7% of female US adults aged 18 and over had hypertension [30]. From the period 1999 to 2001, there was a large APC in the AAMR, with the APC becoming smaller from the period 2007 to 2018. During the same period, the National Health and Nutrition Examination Survey found decreasing rates of hypertension for men and women. With regard to cancer, in the US, age-adjusted incidence rates for all cancers were higher in males compared to females, with an incidence rate ratio of 1.33 [31]. The mortality rates of cancers were also higher in males compared to females overall [32]. The combination of both increased the incidence of hypertension and cancer in the US in males and subsequent mortality, which is reflected in our results from cause of death data, with males having a higher AAMR across the study period.

In advanced economies such as the US, there has been an increasing trend in the number of people in the aging population as lifespans have increased [33]. This has led to a more comorbid society, and as such, there has been an increase in the number of the US population diagnosed with hypertension [34] and cancer. As such, there has been an associated increase in the overall number of patients dying from cancers and associated hypertension. A study analyzed Pennsylvania’s cancer mortality trends (2015–2020) and found that the previously declining rates stalled in 2020 during the COVID-19 pandemic, with 26 counties seeing increases. The 2019 rates strongly predicted 2020 rates, while sociodemographic factors had varying local effects. The top five cancer types remained unchanged, suggesting that the pandemic disrupted progress in reducing cancer deaths, highlighting the need for targeted interventions [35].

With regard to disparities between races, there is a greater disparity between ethnic minorities with higher rates of death from cancer and hypertension compared to the white American population.

Firstly, the data demonstrate that those of Hispanic Latino Origin are more likely to not have health insurance across all ages and states compared to those of white Americans, which reduces access to healthcare for the diagnosis and treatment of hypertension and cancers for this group. Secondly, smoking status is a common risk factor in worsening the clinical outcomes among patients with cancer and hypertension [36,37,38].

Black Americans were found to be less likely to have a successful sustained attempt at quitting smoking lasting one year or stopping smoking entirely, whereas non-Hispanic white Americans were less likely to use pharmacological or behavioral treatments [39]. Thirdly, Hispanic, Latino, and Black African Americans were found to be more likely to live in lower-income areas with higher exposure to air pollution and other environmental pollution in the USA, increasing the risk of developing cancers such as breast, lung, and leukemia [40].

In our results, there was a noticeable increase in the AAMR from the period 2019 to 2021. During this period, the COVID-19 pandemic had a significant effect on patients infected with SARS-CoV-2 and the overall healthcare system. Globally, the priority of healthcare shifted to treating emergency COVID-19 patients as healthcare systems were overwhelmed. Many elective cancer services had been placed on pause or saw a significant reduction in the patient numbers in treatment, increasing the mortality of patients diagnosed with cancer. Cancer patients were unable to readily access primary or secondary healthcare, leading to later diagnoses, worsening outcomes, and increasing mortality [41]. Cancer patients who were immunosuppressed had an increased risk of developing COVID-19 infection and had increased morbidity and mortality compared to the general population [41]. Healthcare providers during this period had to balance the benefits of cancer interventions against the risks of developing an infection of SARS-CoV-2 [42]. In addition, finally, the COVID-19 pandemic disproportionately affected ethnic minority races over the white population. This was reflected in a higher death rate with the African American Black and Hispanic population compared to the non-Hispanic white population [43].

In our study, mortality was higher among males, NH Black individuals, and those living in rural areas. These trends highlight the urgent need for targeted prevention and intervention strategies tailored to high-risk populations. For males, interventions should emphasize regular screening and adherence to treatment for conditions like hypertension, along with promoting heart-healthy behaviors such as reduced sodium intake, increased physical activity, and smoking cessation. Among non-Hispanic Black individuals, culturally tailored approaches that address barriers to care and encourage early detection, dietary improvements, and blood pressure control are essential. In rural communities and southern regions, where access to care may be limited, efforts should focus on improving healthcare availability, increasing awareness about preventive practices, and supporting healthy lifestyle changes. Addressing social determinants of health, including poverty, education, and access to nutritious food, is also critical to reducing disparities and improving outcomes in these vulnerable groups. Moreover, hypertension is strongly associated with elevated body mass index (BMI), with studies showing that individuals who are overweight or obese have a significantly higher risk of developing high blood pressure [44,45]. Excess body fat, particularly visceral fat, can lead to increased sympathetic nervous system activity, insulin resistance, and inflammation, all of which contribute to the development and persistence of hypertension [45]. Even modest reductions in BMI through lifestyle changes such as diet and physical activity have been shown to lower blood pressure and reduce cardiovascular risk [46]. Environmental factors such as air pollution are a major risk factor not only for respiratory and cardiovascular diseases but also for cancer. Recent studies highlight how long-term exposure to pollutants like black carbon may contribute to lung cancer development through inflammation and oxidative stress [47,48].

### Limitations

This study has several limitations. First, the use of ICD codes and death certificates may have led to the misclassification of causes of death due to potential inaccuracies in documentation by healthcare providers. Important details, such as underlying conditions or genetic predispositions, may not have been consistently recorded. Additionally, 2023 data are provisional and subject to further revision by the CDC. The database also lacks detailed clinical information, including vital signs, laboratory findings, imaging results, or genetic testing, which could have enhanced understanding of the underlying mechanisms of cardiac arrest. Furthermore, the absence of data on social determinants of health hinders the assessment of their impact on healthcare access and disparities in outcomes across various racial and ethnic groups.

## 5. Conclusions

Our study is the first to date to comprehensively examine long-term mortality trends among adults with coexisting hypertension and cancer in the United States. Given hypertension’s dual role as both a side effect of cancer therapies and a risk factor for cardiotoxicity, our findings highlight the importance of integrated care approaches that address both oncologic and cardiovascular health. We found that both cancer- and hypertension-related mortality has increased in the United States from 1999 to 2023, with a total of 1,406,107 deaths over the study period. Mortality was higher among males, NH Black Americans or African Americans, and in rural areas. These trends highlight the need for targeted prevention and intervention strategies, particularly for high-risk populations and rural areas. For cancer and hypertension, this includes early screening, lifestyle promotion, and treatment adherence. Addressing social determinants of health is key to reducing disparities and improving outcomes in vulnerable communities.

## Data Availability

All data generated or analyzed during this study are included in this article. Further inquiries can be directed at the corresponding author.

## Supplementary Materials

The following supporting information can be downloaded at https://www.mdpi.com/article/10.3390/medicina61050917/s1. Table S1: Hypertension- and cancer-related deaths stratified by gender and race in the United States; 1999–2023; Table S2: Average annual percentage (95% confidence interval) of hypertension- and cancer-related mortality in the United States; 1999–2023; Table S3: Overall and sex-stratified hypertension- and cancer-related age-adjusted mortality rates per 100,000 in the United States; 1999–2023; Table S4: Race-stratified hypertension- and cancer-related age-adjusted mortality rates per 100,000 in the United States; 1999–2023; Table S5: Hypertension- and cancer-related age-adjusted mortality rates per 100,000 stratified by state in the United States; 1999–2023; Table S6: Hypertension- and cancer-related age-adjusted mortality rates per 100,000 stratified by Census region in the United States; 1999–2023; Table S7: Hypertension- and cancer-related age-adjusted mortality rates per 100,000 stratified by urban and rural status in the United States; 1999–2023; Table S8: Hypertension- and cancer-related crude mortality rates per 100,000 stratified by 10-year age groups in the United States; 1999–2023.
