# Supplementary material for: The Interplay of Cancer and Hypertension: Rising Mortality and Widening Disparities Across the United States (1999–2023)
Source: Medicina (Kaunas). 2025 May 19;61(5):917. doi: 10.3390/medicina61050917 (PMC12113302; doi:10.3390/medicina61050917)
Supplement: Supplementary file 1 [file medicina-61-00917-s001.zip › medicina-3597031-supplementary.pdf]

Supplemental File

Table S1: Hypertension and Cancer related Deaths stratified by Gender and Race in the United States; 1999-2023.

Absolute number of deaths							
**Year**	**Overall**	**Male**	**Female**	**NH Black or African American**	**NH White**	**Hispanic or Latino**	**NH Others**
1999	22209	10018	12191	3868	17109	711	454
2000	32869	15382	17487	5262	25802	1068	643
2001	34541	16266	18275	5471	27036	1218	735
2002	37487	17867	19620	5698	29510	1379	780
2003	39438	18880	20558	6144	30863	1493	858
2004	41081	19814	21267	6103	32339	1610	960
2005	43861	21260	22601	6642	34123	1937	1092
2006	45353	22155	23198	6697	35447	2062	1082
2007	48065	23937	24128	6962	37714	2142	1192
2008	49108	24522	24586	7230	38191	2317	1281
2009	49682	25108	24574	7298	38347	2523	1419
2010	51703	26233	25470	7759	39555	2721	1562
2011	53421	27084	26337	7864	40978	2926	1563
2012	55018	28296	26722	7921	42105	3185	1673
2013	56280	29296	26984	8270	42629	3541	1724
2014	56108	29701	26407	7911	42525	3712	1818
2015	56758	30049	26709	7747	43004	3824	1991
2016	59853	32068	27785	8411	45204	4012	2075
2017	62986	33697	29289	8555	47674	4418	2170
2018	66595	36085	30510	8868	50527	4671	2367
2019	70846	38698	32148	9467	53837	4930	2495
2020	85471	46584	38887	11937	63829	6397	3136
2021	92034	50267	41767	12322	69222	6668	3150
2022	96809	52603	44206	13140	73061	6681	3223
2023	98531	53997	44534	13273	74273	7042	3172

Table S2: Average Annual Percentage (95% confidence interval) of Hypertension and Cancer related Mortality in the United States; 1999-2023.

Variables	Annual Percentage Change (95% CI)
**Gender and Overall popultion**	
**Overall**	1999-2001: APC: 21.05*; 95% CI: 13.18 to 26.63; p < 0.000001.<br>2001-2007: APC: 3.01*; 95% CI: 1.36 to 5.73; p = 0.0036.<br>2007-2018: APC: 0.27; 95% CI: -1.25 to 0.69; p = 0.5187.<br>2018-2021: APC: 12.18*; 95% CI: 9.05 to 14.23; p = 0.0036<br>2021-2023: APC: 1.13; 95% CI: -1.67 to 4.45; p = 0.3687.
**Female**	1999-2001: APC: 19.25*; 95% CI: 11.23 to 25.45; p < 0.000001.<br>2001-2007: APC: 2.66*; 95% CI: 0.97 to 5.09; p = 0.0068.<br>2007-2018: APC: -0.25; 95% CI: -1.92 to 0.22; p = 0.2444.<br>2018-2021: APC: 12.29*; 95% CI: 8.88 to 14.51; p = 0.0080.<br>2021-2023: APC: 0.78; 95% CI: -2.50 to 4.68; p = 0.5479.
**Male**	1999-2001: APC: 22.54*; 95% CI: 13.18 to 29.25; p < 0.000001.<br>2001-2007: APC: 3.23*; 95% CI: 1.56 to 6.03; p = 0.0040.<br>2007-2018: APC: 0.58; 95% CI: -1.11 to 1.01; p = 0.2336.<br>2018-2021: APC: 12.00*; 95% CI: 8.92 to 14.06; p = 0.0024.<br>2021-2023: APC: 1.62; 95% CI: -1.25 to 5.06; p = 0.2484.
**Race/Ethnicity**	
**NH Black or African American**	1999–2001: APC: 16.86 (95% CI: 8.12 to 24.64, p < 0.000001).<br>2001–2010: APC: 0.80 (95% CI: -0.65 to 2.12, p = 0.173).<br>2010–2018: APC: -2.27 (95% CI: -5.44 to 1.59, p = 0.055).<br>2018–2021: APC: 12.17 (95% CI: -2.14 to 14.87, p = 0.079).<br>2021–2023: APC: 1.57 (95% CI: -2.40 to 7.72, p = 0.368).
**NH White**	1999–2001: APC: 22.33 (95% CI: 13.52 to 28.30, p < 0.000001).<br>2001–2007: APC: 3.23 (95% CI: 1.65 to 5.99, p = 0.003).<br>2007–2018: APC: 0.62 (95% CI: -0.97 to 1.04, p = 0.208).<br>2018–2021: APC: 13.12 (95% CI: 9.87 to 15.22, p = 0.002).<br>2021–2023: APC: 1.37 (95% CI: -1.49 to 4.68, p = 0.316).
**Hispanic or Latino**	1999–2005: APC: 8.41 (95% CI: 3.89 to 16.59, p = 0.024).<br>2005–2018: APC: 1.14 (95% CI: -0.75 to 8.38, p = 0.103).<br>2018–2021: APC: 9.16 (95% CI: -0.38 to 11.63, p = 0.062).<br>2021–2023: APC: -0.51 (95% CI: -4.62 to 5.75, p = 0.903).
**NH Others**	1999–2001: APC: 17.98 (95% CI: 5.78 to 29.39, p < 0.000001).<br>2001–2010: APC: 2.02 (95% CI: 0.39 to 3.61, p = 0.028).<br>2010–2018: APC: -1.26 (95% CI: -4.59 to -0.38, p = 0.022).

	2018–2021: APC: 10.04 (95% CI: 6.78 to 12.57, p = 0.006).<br>2021–2023: APC: -3.54 (95% CI: -7.24 to 0.51, p = 0.071).
**Census Region**	
**Northeast**	1999-2001: APC: 18.16*; 95% CI: 6.58 to 25.40; p < 0.000001.<br>2001-2011: APC: 1.87*; 95% CI: 0.42 to 3.49; p = 0.0312.<br>2011-2016: APC: -3.00*; 95% CI: -6.93 to -0.51; p = 0.0296.<br>2016-2023: APC: 4.90*; 95% CI: 3.48 to 6.97; p = 0.0060.
**Midwest**	1999-2001: APC: 20.15*; 95% CI: 12.16 to 26.19; p < 0.00001.<br>2001-2007: APC: 3.59*; 95% CI: 1.79 to 5.46; p = 0.0028.<br>2007-2018: APC: -1.26*; 95% CI: -2.07 to -0.78; p = 0.0016.<br>2018-2021: APC: 11.97*; 95% CI: 8.60 to 14.04; p = 0.0020.<br>2021-2023: APC: -2.13; 95% CI: -5.35 to 1.29; p = 0.2076.
**South**	1999-2001: APC: 28.51*; 95% CI: 13.52 to 38.88; p < 0.000001.<br>2001-2017: APC: 1.84*; 95% CI: 1.18 to 2.34; p < 0.000001.<br>2017-2023: APC: 9.37*; 95% CI: 7.83 to 11.51; p < 0.000001.
**West**	1999-2001: APC: 18.76*; 95% CI: 10.43 to 26.66; p < 0.000001.<br>2001-2007: APC: 3.00*; 95% CI: 1.27 to 5.12; p = 0.0040.<br>2007-2018: APC: 0.27; 95% CI: -1.87 to 0.72; p = 0.5903.<br>2018-2021: APC: 10.51*; 95% CI: 7.62 to 12.56; p = 0.0004.<br>2021-2023: APC: -1.74; 95% CI: -5.01 to 1.79; p = 0.2607.
**2013 Urbanization**	
**Urban**	1999–2001: APC = 19.86 (95% CI: 10.83 to 27.74, p < 0.000001).<br>2001–2007: APC = 2.92 (95% CI: 0.92 to 5.70, p = 0.012).<br>2007–2018: APC = 0.17 (95% CI: -2.89 to 0.69, p = 0.916).<br>2018–2020: APC = 11.73 (95% CI: 5.47 to 16.40, p < 0.000001).
**Rural**	1999–2002: APC = 19.05 (95% CI: 11.16 to 34.07, p < 0.000001).<br>2002–2018: APC = 1.26 (95% CI: 0.19 to 1.76, p = 0.033).<br>2018–2020: APC = 13.58 (95% CI: 4.48 to 18.19, p < 0.000001).
**10-year age groups**	
**35-44 years**	1999–2003: APC = 11.45 (95% CI: 5.98 to 24.43, p = 0.0008).<br>2003–2017: APC = 3.86 (95% CI: -2.26 to 4.57, p = 0.138).

	2017–2023: APC = 7.72 (95% CI: 5.48 to 13.97, p = 0.0036).
**45-54 years**	1999–2009: APC = 6.44 (95% CI: 5.54 to 7.72, p < 0.000001).<br>2009–2018: APC = 1.52 (95% CI: -0.36 to 2.37, p = 0.084).<br>2018–2023: APC = 6.93 (95% CI: 5.06 to 10.09, p < 0.000001).
**55-64 years**	1999–2001: APC = 15.91 (95% CI: 7.33 to 23.64, p < 0.000001).<br>2001–2007: APC = 3.93 (95% CI: 1.63 to 5.73, p = 0.0152).<br>2007–2018: APC = 1.67 (95% CI: -0.74 to 2.25, p = 0.096).<br>2018–2021: APC = 11.50 (95% CI: 8.85 to 13.49, p < 0.000001).<br>2021–2023: APC = -1.70 (95% CI: -4.74 to 1.46, p = 0.252).
**65-74 years**	1999–2001: APC = 23.25 (95% CI: 12.25 to 32.73, p < 0.000001).<br>2001–2018: APC = 0.61 (95% CI: -0.20 to 1.01, p = 0.072).<br>2018–2021: APC = 11.32 (95% CI: 0.03 to 13.27, p = 0.0496).<br>2021–2023: APC = 2.18 (95% CI: -1.75 to 7.71, p = 0.166).
**75-84 years**	1999–2001: APC = 21.09 (95% CI: 9.56 to 29.44, p < 0.000001).<br>2001–2007: APC = 3.05 (95% CI: 0.09 to 6.09, p = 0.046).<br>2007–2017: APC = -0.35 (95% CI: -3.41 to 5.43, p = 0.360).<br>2017–2023: APC = 7.49 (95% CI: 5.67 to 9.77, p = 0.0008).
**85+ years**	1999–2001: APC = 23.85 (95% CI: 13.39 to 31.29, p < 0.000001).<br>2001–2007: APC = 3.36 (95% CI: 1.40 to 6.52, p = 0.0056).<br>2007–2018: APC = 0.17 (95% CI: -1.46 to 0.65, p = 0.745).<br>2018–2021: APC = 13.62 (95% CI: 10.05 to 16.00, p = 0.0008).<br>2021–2023: APC = -0.89 (95% CI: -4.16 to 2.84, p = 0.541).

Table S3: Overall and Sex stratified Hypertension and Cancer related Age Adjusted Mortality Rates per 100,000 in the United States; 1999-2023.

**Age Adjusted Mortality Rate per 100,000 (95% CI)**			
**Year**	**Overall**	**Male**	**Female**
1999	12.59 (12.42 - 12.76)	14.52 (14.23 - 14.81)	11.35 (11.14 - 11.55)
2000	18.42 (18.22 - 18.62)	22.18 (21.82 - 22.54)	16.01 (15.77 - 16.25)
2001	19.06 (18.86 - 19.26)	22.91 (22.55 - 23.27)	16.59 (16.35 - 16.83)
2002	20.37 (20.17 - 20.58)	24.75 (24.38 - 25.12)	17.59 (17.34 - 17.83)
2003	21.07 (20.87 - 21.28)	25.62 (25.25 - 25.99)	18.19 (17.94 - 18.44)
2004	21.64 (21.44 - 21.85)	26.3 (25.93 - 26.67)	18.62 (18.37 - 18.87)
2005	22.69 (22.47 - 22.9)	27.46 (27.09 - 27.84)	19.49 (19.23 - 19.74)
2006	23.02 (22.81 - 23.24)	27.93 (27.56 - 28.3)	19.69 (19.43 - 19.94)
2007	23.91 (23.7 - 24.13)	29.5 (29.12 - 29.88)	20.13 (19.87 - 20.39)
2008	23.91 (23.7 - 24.12)	29.39 (29.02 - 29.76)	20.19 (19.94 - 20.45)
2009	23.7 (23.49 - 23.91)	29.33 (28.96 - 29.7)	19.83 (19.58 - 20.08)
2010	24.23 (24.02 - 24.44)	30.03 (29.66 - 30.4)	20.3 (20.04 - 20.55)
2011	24.34 (24.13 - 24.55)	29.83 (29.47 - 30.19)	20.47 (20.22 - 20.72)
2012	24.41 (24.2 - 24.62)	30.17 (29.82 - 30.53)	20.32 (20.07 - 20.57)
2013	24.35 (24.15 - 24.56)	30.29 (29.94 - 30.64)	20.15 (19.91 - 20.39)
2014	23.7 (23.51 - 23.9)	29.75 (29.41 - 30.1)	19.28 (19.05 - 19.52)
2015	23.42 (23.22 - 23.61)	29.32 (28.99 - 29.66)	19.12 (18.88 - 19.35)
2016	24.14 (23.94 - 24.33)	30.45 (30.11 - 30.79)	19.55 (19.32 - 19.78)
2017	24.75 (24.56 - 24.95)	31.11 (30.77 - 31.44)	20.1 (19.87 - 20.34)
2018	25.55 (25.35 - 25.74)	32.27 (31.93 - 32.61)	20.52 (20.29 - 20.75)
2019	26.52 (26.33 - 26.72)	33.74 (33.4 - 34.08)	21.16 (20.92 - 21.39)
2020	31.39 (31.18 - 31.61)	39.73 (39.37 - 40.1)	25.16 (24.91 - 25.42)
2021	34.69 (34.47 - 34.92)	43.64 (43.25 - 44.03)	27.98 (27.71 - 28.26)
2022	34.83 (34.61 - 35.05)	43.91 (43.53 - 44.29)	28.08 (27.82 - 28.35)
2023	35.49 (35.27 - 35.71)	45.23 (44.84 - 45.62)	28.33 (28.06 - 28.59)

Table S4: Race stratified Hypertension and Cancer related Age Adjusted Mortality Rates per 100,000 in the United States; 1999-2023.

Age Adjusted Mortality Rate per 100,000 (95% CI)				
Year	NH Black or African American	NH White	Hispanic or Latino	NH Others
1999	26.51 (25.66 - 27.35)	11.42 (11.25 - 11.59)	8.76 (8.09 - 9.43)	10.27 (9.29 - 11.26)
2000	35.94 (34.95 - 36.92)	17.06 (16.85 - 17.27)	12.67 (11.88 - 13.45)	13.85 (12.74 - 14.96)
2001	36.65 (35.67 - 37.63)	17.7 (17.49 - 17.91)	13.56 (12.78 - 14.35)	14.56 (13.47 - 15.65)
2002	37.7 (36.71 - 38.69)	19.11 (18.89 - 19.33)	14.71 (13.9 - 15.51)	14.91 (13.83 - 16)
2003	39.75 (38.74 - 40.75)	19.78 (19.56 - 20)	14.83 (14.06 - 15.61)	15.52 (14.45 - 16.59)
2004	38.69 (37.7 - 39.68)	20.49 (20.27 - 20.72)	15.27 (14.5 - 16.04)	16.37 (15.3 - 17.44)
2005	40.94 (39.94 - 41.95)	21.35 (21.13 - 21.58)	17.36 (16.56 - 18.15)	17.45 (16.39 - 18.52)
2006	40.14 (39.16 - 41.12)	21.85 (21.62 - 22.08)	17.62 (16.83 - 18.41)	16.07 (15.09 - 17.06)
2007	40.79 (39.82 - 41.77)	22.87 (22.63 - 23.1)	17.47 (16.71 - 18.24)	16.97 (15.98 - 17.96)
2008	41.08 (40.11 - 42.04)	22.81 (22.58 - 23.03)	17.63 (16.89 - 18.37)	17.34 (16.37 - 18.32)
2009	40.22 (39.27 - 41.16)	22.55 (22.32 - 22.77)	18.25 (17.51 - 18.98)	17.91 (16.95 - 18.87)
2010	41.69 (40.74 - 42.65)	22.93 (22.71 - 23.16)	18.82 (18.09 - 19.55)	19.04 (18.07 - 20.01)
2011	40.43 (39.51 - 41.35)	23.29 (23.07 - 23.52)	18.86 (18.15 - 19.56)	17.36 (16.48 - 18.25)
2012	39.5 (38.61 - 40.4)	23.42 (23.19 - 23.65)	19.4 (18.71 - 20.1)	17.72 (16.85 - 18.58)
2013	39.63 (38.75 - 40.51)	23.28 (23.06 - 23.51)	20.34 (19.65 - 21.02)	16.86 (16.05 - 17.67)
2014	36.4 (35.57 - 37.22)	22.85 (22.63 - 23.07)	20 (19.34 - 20.67)	16.68 (15.9 - 17.46)
2015	34.37 (33.58 - 35.16)	22.76 (22.54 - 22.98)	19.25 (18.63 - 19.88)	16.95 (16.19 - 17.71)
2016	36.34 (35.54 - 37.14)	23.53 (23.31 - 23.75)	19.47 (18.85 - 20.09)	16.77 (16.03 - 17.5)

2017	35.55 (34.77 - 36.33)	24.32 (24.1 - 24.55)	20.09 (19.48 - 20.7)	16.49 (15.78 - 17.19)
2018	35.69 (34.92 - 36.45)	25.3 (25.08 - 25.52)	20.36 (19.76 - 20.96)	17.09 (16.39 - 17.79)
2019	36.91 (36.15 - 37.67)	26.45 (26.23 - 26.68)	20.71 (20.12 - 21.31)	17.05 (16.37 - 17.73)
2020	45.14 (44.31 - 45.97)	30.98 (30.74 - 31.22)	25.69 (25.04 - 26.33)	20.58 (19.85 - 21.31)
2021	47.68 (46.81 - 48.55)	35.09 (34.82 - 35.35)	26.45 (25.8 - 27.11)	21.68 (20.92 - 22.45)
2022	49.24 (48.37 - 50.1)	35.32 (35.06 - 35.58)	25.23 (24.6 - 25.85)	20.66 (19.94 - 21.38)
2023	50.04 (49.17 - 50.92)	35.88 (35.62 - 36.14)	26.7 (26.06 - 27.34)	20.43 (19.71 - 21.15)

Table S5: Hypertension and Cancer related Age Adjusted Mortality Rates per 100,000 stratified by States in the United States; 1999-2023.

**Age Adjusted Mortality Rate per 100,000 (95% CI)**		
**States**	**1999-2020**	**2021-2023**
Alabama	19.46 (19.14 - 19.77)	28.77 (27.83 - 29.71)
Alaska	17.13 (16.04 - 18.23)	18.07 (15.69 - 20.45)
Arizona	13.12 (12.89 - 13.34)	24.25 (23.55 - 24.94)
Arkansas	24.82 (24.37 - 25.27)	46.89 (45.35 - 48.44)
California	29.01 (28.87 - 29.16)	33.25 (32.87 - 33.64)
Colorado	22.28 (21.92 - 22.65)	45.54 (44.33 - 46.75)
Connecticut	17.82 (17.48 - 18.15)	15.52 (14.72 - 16.31)
Delaware	18.62 (17.92 - 19.32)	52.19 (49.5 - 54.88)
District of Columbia	34.5 (33.24 - 35.75)	40.81 (37.33 - 44.3)
Florida	15.93 (15.8 - 16.06)	28.46 (28.06 - 28.86)
Georgia	19.78 (19.53 - 20.03)	37.62 (36.82 - 38.41)
Hawaii	21.07 (20.48 - 21.66)	21.52 (20.12 - 22.93)
Idaho	19.68 (19.1 - 20.26)	42.55 (40.59 - 44.51)
Illinois	18.85 (18.66 - 19.05)	21.24 (20.72 - 21.76)
Indiana	24.83 (24.52 - 25.14)	35.79 (34.86 - 36.72)
Iowa	22.99 (22.59 - 23.39)	34.09 (32.83 - 35.35)
Kansas	16.19 (15.82 - 16.56)	23.73 (22.6 - 24.87)
Kentucky	26.24 (25.85 - 26.64)	47.13 (45.83 - 48.43)
Louisiana	25.44 (25.06 - 25.83)	50.88 (49.52 - 52.23)
Maine	16.09 (15.59 - 16.59)	27 (25.4 - 28.59)
Maryland	28.92 (28.56 - 29.29)	51.49 (50.34 - 52.65)
Massachusetts	15.6 (15.37 - 15.83)	16.97 (16.36 - 17.57)
Michigan	22.58 (22.35 - 22.81)	28.37 (27.71 - 29.02)
Minnesota	28.95 (28.59 - 29.32)	55.77 (54.53 - 57.01)
Mississippi	41.68 (41.07 - 42.28)	83.58 (81.43 - 85.73)
Missouri	20.9 (20.61 - 21.19)	26.28 (25.47 - 27.09)
Montana	16.2 (15.59 - 16.8)	29.56 (27.58 - 31.53)
Nebraska	34.01 (33.35 - 34.67)	64.71 (62.38 - 67.03)
Nevada	12.61 (12.24 - 12.99)	34.65 (33.27 - 36.03)
New Hampshire	20.14 (19.53 - 20.74)	21.94 (20.44 - 23.44)

New Jersey	23.36 (23.11 - 23.61)	24.82 (24.18 - 25.46)
New Mexico	16.21 (15.76 - 16.66)	27.97 (26.55 - 29.39)
New York	19.99 (19.83 - 20.15)	31.03 (30.55 - 31.51)
North Carolina	23.25 (23 - 23.5)	32.96 (32.26 - 33.67)
North Dakota	29.6 (28.63 - 30.57)	37.26 (34.46 - 40.06)
Ohio	34 (33.74 - 34.26)	30.48 (29.85 - 31.1)
Oklahoma	40.42 (39.9 - 40.94)	98.56 (96.54 - 100.59)
Oregon	24.04 (23.66 - 24.43)	45.32 (44.05 - 46.6)
Pennsylvania	23.34 (23.15 - 23.54)	26.92 (26.38 - 27.46)
Rhode Island	28.96 (28.19 - 29.73)	47.9 (45.4 - 50.4)
South Carolina	22.48 (22.13 - 22.83)	64.04 (62.68 - 65.41)
South Dakota	25.3 (24.48 - 26.13)	41.32 (38.62 - 44.03)
Tennessee	26.79 (26.46 - 27.11)	43.23 (42.23 - 44.23)
Texas	30.36 (30.17 - 30.56)	44.95 (44.39 - 45.51)
Utah	9.49 (9.13 - 9.85)	14.92 (13.88 - 15.96)
Vermont	28.75 (27.73 - 29.77)	33.41 (30.78 - 36.05)
Virginia	17.82 (17.58 - 18.07)	32.41 (31.62 - 33.19)
Washington	20 (19.72 - 20.28)	29.52 (28.71 - 30.32)
West Virginia	32.43 (31.82 - 33.04)	41.54 (39.76 - 43.32)
Wisconsin	19.46 (19.18 - 19.74)	36.47 (35.5 - 37.43)
Wyoming	20.41 (19.42 - 21.4)	48.85 (45.15 - 52.55)
Total	23.41 (23.37 - 23.45)	35.02 (34.89 - 35.15)

Table S6: Hypertension and Cancer related Age Adjusted Mortality Rates per 100,000 stratified by Census Region in the United States; 1999-2023.

Age Adjusted Mortality Rate per 100,000 (95% CI)				
**Year**	**Northeast**	**Midwest**	**South**	**West**
1999	12.56 (12.2 - 12.92)	14.22 (13.86 - 14.58)	11.11 (10.84 - 11.37)	13.27 (12.89 - 13.65)
2000	18.41 (17.98 - 18.85)	19.97 (19.55 - 20.4)	17.31 (16.98 - 17.63)	18.42 (17.97 - 18.87)
2001	18.27 (17.85 - 18.7)	21.06 (20.62 - 21.49)	18.01 (17.68 - 18.34)	19.32 (18.87 - 19.78)
2002	20.02 (19.57 - 20.46)	22.29 (21.85 - 22.74)	19.51 (19.17 - 19.85)	20.01 (19.55 - 20.46)
2003	19.34 (18.9 - 19.77)	23.7 (23.25 - 24.16)	20.25 (19.9 - 20.59)	21.2 (20.74 - 21.67)
2004	20.15 (19.71 - 20.6)	24.15 (23.69 - 24.61)	20.58 (20.24 - 20.93)	22.12 (21.65 - 22.59)
2005	20.78 (20.34 - 21.23)	25.22 (24.76 - 25.69)	21.73 (21.38 - 22.08)	23.29 (22.81 - 23.77)
2006	20.93 (20.49 - 21.38)	26.1 (25.63 - 26.57)	22.34 (21.98 - 22.69)	22.73 (22.26 - 23.19)
2007	21.68 (21.23 - 22.13)	27.05 (26.58 - 27.53)	23.19 (22.84 - 23.54)	23.64 (23.17 - 24.11)
2008	21.58 (21.13 - 22.03)	26.99 (26.52 - 27.46)	23.18 (22.83 - 23.53)	23.96 (23.49 - 24.42)
2009	21.26 (20.82 - 21.7)	26.16 (25.7 - 26.61)	23.43 (23.08 - 23.77)	23.7 (23.24 - 24.16)
2010	23.23 (22.76 - 23.69)	25.49 (25.04 - 25.94)	24.1 (23.75 - 24.45)	23.95 (23.5 - 24.41)
2011	23.33 (22.87 - 23.79)	26.07 (25.61 - 26.52)	23.8 (23.46 - 24.14)	24.19 (23.74 - 24.64)
2012	22.51 (22.06 - 22.96)	25.74 (25.29 - 26.18)	24.35 (24.01 - 24.7)	24.71 (24.27 - 25.16)
2013	21.72 (21.28 - 22.16)	24.68 (24.25 - 25.12)	25.29 (24.95 - 25.63)	24.69 (24.25 - 25.13)
2014	21.14 (20.71 - 21.57)	23.71 (23.29 - 24.13)	24.43 (24.1 - 24.76)	24.48 (24.04 - 24.91)
2015	20.64 (20.22 - 21.06)	24.27 (23.85 - 24.7)	24.1 (23.77 - 24.42)	23.59 (23.18 - 24.01)
2016	20.11 (19.69 - 20.52)	24.38 (23.96 - 24.8)	25.74 (25.4 - 26.07)	24.47 (24.05 - 24.89)
2017	20.61 (20.2 - 21.02)	24.32 (23.91 - 24.74)	27.3 (26.96 - 27.64)	24.27 (23.86 - 24.69)

2018	20.89 (20.48 - 21.3)	24.03 (23.62 - 24.44)	28.68 (28.34 - 29.02)	25.5 (25.09 - 25.92)
2019	21.1 (20.7 - 21.51)	24.98 (24.57 - 25.4)	30.27 (29.93 - 30.62)	26.1 (25.69 - 26.52)
2020	26.12 (25.67 - 26.57)	29.99 (29.54 - 30.44)	35.37 (35 - 35.74)	30.27 (29.83 - 30.71)
2021	26.09 (25.63 - 26.54)	33.32 (32.84 - 33.81)	40.28 (39.88 - 40.68)	33.75 (33.27 - 34.22)
2022	26.84 (26.39 - 27.29)	31.72 (31.26 - 32.18)	42.13 (41.73 - 42.52)	31.85 (31.4 - 32.3)
2023	26.4 (25.96 - 26.85)	31.86 (31.39 - 32.32)	43.77 (43.36 - 44.17)	32.4 (31.95 - 32.85)

Table S7: Hypertension and Cancer related Age Adjusted Mortality Rates per 100,000 stratified by Urban-Rural status in the United States; 1999-2023.

Age Adjusted Mortality Rate per 100,000 (95% CI)		
**Year**	**Urban**	**Rural**
1999	12.53 (12.35 - 12.72)	12.79 (12.41 - 13.17)
2000	18.21 (17.99 - 18.43)	19.32 (18.85 - 19.78)
2001	18.75 (18.53 - 18.98)	20.41 (19.93 - 20.89)
2002	19.93 (19.7 - 20.15)	22.38 (21.88 - 22.88)
2003	20.52 (20.29 - 20.75)	23.6 (23.09 - 24.11)
2004	21.01 (20.78 - 21.24)	24.49 (23.98 - 25.01)
2005	22.05 (21.82 - 22.28)	25.55 (25.02 - 26.08)
2006	22.34 (22.11 - 22.58)	25.93 (25.41 - 26.46)
2007	23.1 (22.86 - 23.33)	27.37 (26.84 - 27.91)
2008	23.16 (22.93 - 23.39)	27.43 (26.9 - 27.97)
2009	22.9 (22.67 - 23.12)	27.36 (26.83 - 27.89)
2010	23.71 (23.48 - 23.94)	26.74 (26.22 - 27.26)
2011	23.78 (23.55 - 24)	26.93 (26.41 - 27.45)
2012	23.8 (23.57 - 24.02)	27.47 (26.95 - 27.99)
2013	23.8 (23.58 - 24.02)	27.15 (26.63 - 27.66)
2014	23.1 (22.89 - 23.32)	26.61 (26.1 - 27.11)
2015	22.54 (22.33 - 22.75)	27.59 (27.08 - 28.1)
2016	23.2 (22.99 - 23.41)	28.79 (28.27 - 29.31)
2017	23.74 (23.53 - 23.96)	29.77 (29.24 - 30.29)
2018	24.51 (24.29 - 24.72)	30.76 (30.23 - 31.29)
2019	25.36 (25.15 - 25.57)	32.63 (32.09 - 33.17)
2020	29.99 (29.76 - 30.22)	38.71 (38.13 - 39.3)

Table S8: Hypertension and Cancer related Crude Mortality Rates per 100,000 stratified by 10-year age groups in the United States; 1999-2023.

**Crude Mortality Rate per 100,000 (95% CI)**						
**Year**	**25-34 years**	**35-44 years**	**55-64 years**	**65-74 years**	**75-84 years**	**85+ years**
1999	Unreliable (0.02 - 0.06)	0.26 (0.22 - 0.31)	1.82 (1.69 - 1.96)	30.06 (29.27 - 30.85)	67.8 (66.34 - 69.26)	131.9 (128.4 - 135.39)
2000	Unreliable (0.02 - 0.07)	0.29 (0.24 - 0.34)	2.28 (2.12 - 2.43)	41.4 (40.47 - 42.33)	102.68 (100.9 - 104.47)	207.54 (203.21 - 211.88)
2001	0.05 (0.03 - 0.08)	0.35 (0.3 - 0.41)	2.34 (2.19 - 2.49)	43.75 (42.79 - 44.71)	104.97 (103.18 - 106.76)	210.85 (206.52 - 215.19)
2002	Unreliable (0.01 - 0.05)	0.32 (0.27 - 0.37)	2.49 (2.33 - 2.64)	46.87 (45.88 - 47.86)	111.83 (110 - 113.66)	231.87 (227.36 - 236.39)
2003	0.08 (0.06 - 0.12)	0.44 (0.38 - 0.5)	2.6 (2.44 - 2.76)	47.1 (46.11 - 48.09)	114.64 (112.8 - 116.49)	242.56 (237.99 - 247.12)
2004	Unreliable (0.03 - 0.07)	0.39 (0.34 - 0.45)	2.77 (2.61 - 2.93)	48.49 (47.49 - 49.49)	119.25 (117.37 - 121.12)	246.27 (241.7 - 250.83)
2005	0.05 (0.03 - 0.08)	0.42 (0.36 - 0.48)	3 (2.83 - 3.16)	49.92 (48.91 - 50.93)	124.73 (122.81 - 126.64)	259.65 (255.04 - 264.26)
2006	0.09 (0.06 - 0.12)	0.46 (0.4 - 0.52)	3.16 (2.99 - 3.33)	50.56 (49.56 - 51.57)	125.94 (124.02 - 127.86)	263.3 (258.74 - 267.86)
2007	0.1 (0.07 - 0.13)	0.47 (0.4 - 0.53)	3.19 (3.02 - 3.35)	51.68 (50.67 - 52.68)	131.27 (129.31 - 133.23)	275.66 (271.08 - 280.24)
2008	0.07 (0.05 - 0.1)	0.5 (0.43 - 0.57)	3.53 (3.35 - 3.7)	51.42 (50.44 - 52.41)	130.83 (128.87 - 132.79)	273.91 (269.41 - 278.41)
2009	0.08 (0.06 - 0.11)	0.47 (0.4 - 0.54)	3.84 (3.66 - 4.02)	50.56 (49.61 - 51.52)	128.51 (126.56 - 130.45)	273.69 (269.27 - 278.12)
2010	0.1 (0.07 - 0.13)	0.54 (0.47 - 0.61)	3.83 (3.65 - 4.02)	51.75 (50.79 - 52.71)	130.43 (128.47 - 132.39)	281.35 (276.92 - 285.79)
2011	0.09 (0.06 - 0.12)	0.53 (0.46 - 0.6)	3.86 (3.68 - 4.04)	52.01 (51.06 - 52.95)	130.96 (129 - 132.91)	278.62 (274.3 - 282.94)
2012	0.1 (0.08 - 0.14)	0.57 (0.5 - 0.64)	3.92 (3.73 - 4.1)	51.78 (50.87 - 52.69)	130.45 (128.51 - 132.39)	281.77 (277.49 - 286.06)
2013	0.09 (0.06 - 0.12)	0.64 (0.56 - 0.72)	4.24 (4.04 - 4.43)	51.39 (50.51 - 52.28)	130.75 (128.81 - 132.68)	276.62 (272.43 - 280.81)
2014	0.09 (0.07 - 0.13)	0.66 (0.58 - 0.74)	3.96 (3.77 - 4.14)	50.08 (49.22 - 50.93)	126.03 (124.15 - 127.91)	269.3 (265.2 - 273.4)
2015	0.08 (0.06 - 0.11)	0.58 (0.5 - 0.65)	3.86 (3.68 - 4.05)	49.46 (48.63 - 50.29)	124.54 (122.69 - 126.39)	269.96 (265.9 - 274.02)
2016	0.11 (0.08 - 0.15)	0.64 (0.56 - 0.72)	4.36 (4.16 - 4.56)	51.4 (50.57 - 52.23)	127.86 (126 - 129.72)	272.84 (268.79 - 276.89)
2017	0.1 (0.07 - 0.13)	0.68 (0.6 - 0.76)	4.28 (4.08 - 4.47)	52.77 (51.94 - 53.6)	128.48 (126.65 - 130.31)	286.72 (282.59 - 290.85)

2018	0.11 (0.08 - 0.15)	0.7 (0.61 - 0.78)	4.4 (4.2 - 4.6)	54.66 (53.83 - 55.49)	133.48 (131.66 - 135.31)	290.69 (286.56 - 294.82)
2019	0.12 (0.09 - 0.16)	0.79 (0.71 - 0.88)	4.34 (4.14 - 4.54)	57.13 (56.3 - 57.97)	136.52 (134.71 - 138.33)	309 (304.76 - 313.23)
2020	0.13 (0.1 - 0.16)	0.9 (0.81 - 0.99)	5.14 (4.92 - 5.36)	67.2 (66.31 - 68.09)	162.95 (161 - 164.9)	361.44 (356.87 - 366)
2021	0.15 (0.11 - 0.19)	1 (0.91 - 1.1)	5.38 (5.15 - 5.61)	72.34 (71.43 - 73.25)	180.15 (178.09 - 182.22)	415.45 (410.28 - 420.61)
2022	0.15 (0.12 - 0.19)	0.98 (0.89 - 1.07)	5.76 (5.53 - 6)	75.4 (74.48 - 76.33)	180.7 (178.71 - 182.69)	402.74 (397.85 - 407.62)
2023	0.16 (0.12 - 0.2)	1.02 (0.92 - 1.11)	5.88 (5.64 - 6.12)	76.01 (75.08 - 76.94)	189.24 (187.2 - 191.28)	404.99 (400.09 - 409.89)
